# A polyphenol-enriched diet and *Ascaris suum* infection modulate mucosal immune responses and gut microbiota composition in pigs

**DOI:** 10.1371/journal.pone.0186546

**Published:** 2017-10-13

**Authors:** Andrew R. Williams, Lukasz Krych, Hajar Fauzan Ahmad, Peter Nejsum, Kerstin Skovgaard, Dennis S. Nielsen, Stig M. Thamsborg

**Affiliations:** 1 Department of Veterinary and Animal Sciences, Faculty of Health and Medical Sciences, University of Copenhagen, Frederiksberg, Denmark; 2 Department of Food Sciences, Faculty of Science, University of Copenhagen, Frederiksberg, Denmark; 3 Department of Industrial Biotechnology, Faculty of Industrial Sciences and Technology, University Malaysia Pahang, Pahang, Malaysia; 4 Department of Clinical Medicine, Faculty of Health, Aarhus University, Aarhus, Denmark; 5 Section for Immunology and Vaccinology, Technical University of Denmark, Frederiksberg, Denmark; UMASS Medical School, UNITED STATES

## Abstract

Polyphenols are a class of bioactive plant secondary metabolites that are thought to have beneficial effects on gut health, such as modulation of mucosal immune and inflammatory responses and regulation of parasite burdens. Here, we examined the interactions between a polyphenol-rich diet supplement and infection with the enteric nematode *Ascaris suum* in pigs. Pigs were fed either a basal diet or the same diet supplemented with grape pomace (GP), an industrial by-product rich in polyphenols such as oligomeric proanthocyanidins. Half of the animals in each group were then inoculated with *A*. *suum* for 14 days to assess parasite establishment, acquisition of local and systemic immune responses and effects on the gut microbiome. Despite *in vitro* anthelmintic activity of GP-extracts, numbers of parasite larvae in the intestine were not altered by GP-supplementation. However, the bioactive diet significantly increased numbers of eosinophils induced by *A*. *suum* infection in the duodenum, jejunum and ileum, and modulated gene expression in the jejunal mucosa of infected pigs. Both GP-supplementation and *A*. *suum* infection induced significant and apparently similar changes in the composition of the prokaryotic gut microbiota, and both also decreased concentrations of isobutyric and isovaleric acid (branched-chain short chain fatty acids) in the colon. Our results demonstrate that while a polyphenol-enriched diet in pigs may not directly influence *A*. *suum* establishment, it significantly modulates the subsequent host response to helminth infection. Our results suggest an influence of diet on immune function which may potentially be exploited to enhance immunity to helminths.

## Introduction

The effect of diet on gastrointestinal health and immune function in animals is an area of intense interest, in part due to the need to increase health and production without recourse to synthetic antibiotics and anti-parasitic drugs. Gastrointestinal helminths are one of the most ubiquitous pathogens in both humans and livestock worldwide. More than a billion people are estimated to be infected with soil-transmitted helminths such as *Ascaris lumbricoides*, *Trichuris trichiura* and hookworms, and helminths are present in virtually all livestock production systems of economic importance, causing reduced growth rates and feed conversion ratios in both ruminants and monogastric animals such as pigs and horses [1, 2]. Helminth infections are routinely treated with a small number of synthetic drugs, and this heavy reliance on a limited arsenal of anthelmintics is an increasingly important issue in livestock production, due to reports of drug-resistance in nematodes of most important livestock species, as well as consumer demand for animal products delivered with a minimum of synthetic chemical inputs [2–5].

The use of bioactive dietary components to improve animal health and performance has gained considerable traction in recent years, due to increasing pressure to reduce antibiotic and anthelmintic use.

The provision of diets rich in plant secondary compounds such as polyphenols has of been particular interest. Common polyphenols include flavanols, ellagitannins and proanthocyanidins (PAC; *syn*-condensed tannins). PAC are commonly found in nuts, berries and grapes and consist of hetero-polymeric forms of flavan-3-ols, with the degree of polymerization (chain length) thought to be an important factor in their bioactivity [6, 7]. The most common flavan-3-ol monomeric subunits are either catechin and its *cis*-isomer epicatechin, which give rise to procyanidin-type PAC, and gallocatechin and epigallocatechin, which give rise to prodelphinidin-type PAC [8] (S1 Fig). Antimicrobial effects of polyphenols are well-known; PAC- or ellagitannin-containing diets have been shown to effectively reduce helminth infection in sheep, goats, and cattle [9–11], and PAC-rich supplements have also shown promise in controlling gut pathogens in pigs such as *Escherichia coli* [12, 13].

In addition to direct antimicrobial properties, dietary polyphenols can also have wide-ranging effect on many aspects of animal physiology. For example, both procyanidin and prodelphinidin-type PAC may modulate immune function by acting as T-cell agonists and regulating inflammatory responses in antigen-presenting cells [14, 15]. Furthermore, grape seed products which are rich in oligomeric PAC may modify gut microbiota composition and metabolic function [16], and decrease oxidative stress and inflammatory responses in the gut mucosa [17, 18]. Helminths normally induce strong local immune responses at the site of infection and can markedly alter normal microbiota composition and host metabolism [19, 20]. Therefore, in addition to direct anti-parasitic effects of dietary polyphenols, a key question of interest is whether they regulate host immune responses and gut microbiota during helminth infection.

*Ascaris suum* is a parasite of worldwide importance in pig production, and also serves as a valuable infection model for studying the influence of dietary factors on immunological function due to the potent immune reactions that are generated following primary inoculations. Primary infections result in a high establishment of larvae which can cause significant inflammation in the intestine, liver and lungs, as well as inhibiting animal performance [21–24]. Between 14–17 days following larval inoculation, parasite-specific antibodies sharply increase and a strong localised immune responses develop in the gut which remove the majority of the larvae, however a small residual adult worm population may develop. After repeated infections, robust protective immunity is eventually developed whereby most incoming larvae are expelled after ingestion without beginning the hepatic-tracheal migration. Whilst the exact mechanisms leading to larval expulsion and immunity are not fully clear, a transient rise in Th2-type cytokines, mucus secretion, and local eosinophilia and mastocytosis around day 10–14 post-larval inoculation have all been observed and proposed to play a role [25–27].

Recently, we demonstrated that purified PAC molecules have strong anthelmintic effects *in vitro* against *A*. *suum* [6, 28], as well as other potential beneficial effects on health and immune function, such as direct activation of porcine immune cells [29], and enhancement of Th2-type responses to helminth antigens in human dendritic cells [30]. Therefore, it is possible that dietary components such as PAC and other polyphenols may exert a strong influence on the interactions between helminths and their hosts. In the current study, we hypothesised that a polyphenol-rich diet supplement may modulate host responses to helminth infection *in vivo*, potentially inhibiting larval establishment and concurrently stimulating acquisition of Th2-type mucosal immune function. Grape pomace (GP) is a by-product of wine production that is cheap, easily sourced and particularly rich in oligomeric PAC, and has been shown to have beneficial effects on pig growth and gut health [31]. Here, we investigated specifically whether supplementing diets with GP could 1) reduce the establishment of *A*. *suum* in young, growing pigs and 2) modulate acquisition of immune responses and gut microbiota composition during the establishment of the infection.

## Results

### Anthelmintic activity of grape pomace against *Ascaris suum in vitro* and *in vivo*

**Fig 1:**
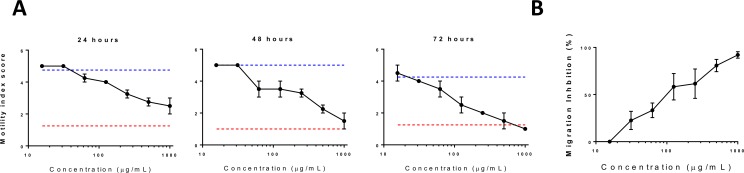
*In vitro* anthelmintic activity of grape pomace (GP) extract against *Ascaris suum* larvae. **A)** Inhibition of *Ascaris suum* larval motility by acetone-water GP extract. Larvae were exposed to varying concentrations of the extract, culture medium only (blue line) or 25 μg/mL levamisole (red line) for 24, 48 or 72 hours. **B)** Inhibition of migration of *Ascaris suum* larvae after 24 hours incubation in acetone-water extract of GP. Inhibition is expressed as a percentage relative to larvae exposed only to culture medium.

We have previously observed that purified PAC molecules have direct anthelmintic effects *in vitro* against *A*. *suum* larvae in a polymerisation-dependent manner [6]. To explore whether this could be translated to the *in vivo* situation, we selected GP as a PAC-containing dietary supplement due to its ready availability and reported beneficial effects on pig performance [31]. Analysis of PAC in the GP is presented in Table 1. The GP contained 45g PAC / kg, with the PAC mostly comprised of catechin and epicatechin, with a mean degree of polymerisation (i.e. average polymer size) of 9.8. Furthermore, around 10% of the flavan-3-ol units were gallyoated, i.e. had a galloyl group attached to the C-ring of the monomeric sub-unit. To assess *in vitro* anthelmintic activity of the GP against *A*. *suum* larvae, we prepared an acetone/water extract, which contained around 10% PAC (v/v). Consistent with our previous studies using PAC-rich extracts from a range of plants [6], acetone-water extracts prepared from GP showed dose-dependent *in vitro* anthelmintic activity against *A*. *suum* L3. Larvae exposed to the GP extract showed a progressive decrease in motility over 72 hours, and their migratory ability was impaired after 24 hours incubation in the extract (Fig 1A and 1B).

**Table 1:** Analysed composition of proanthocyanidins present in grape pomace.

Degree of polymerization	% procyanidin	% prodelphinidin	% galloylated	*cis*: *trans* ratio
9.8	85.5	14.5	10	87.5: 12.5

We next investigated parasite establishment and acquisition of immune responses in pigs fed diets supplemented with GP. Two groups of pigs (each n = 12) were fed either a normal basal diet or an enriched diet containing 5% GP. The inclusion rate in the diet was chosen based on previous studies examining the effects of GP or similar polyphenol-rich products on gut health in pigs [17, 18], as well as extrapolating from our current and previous *in vitro* data the concentration of PAC that would need to be present in the intestine to exert bioactivity [6]. After 7 days acclimatisation to the diet, half of the pigs in each dietary group (n = 6) were infected with *A*. *suum* and the infection allowed to proceed for 14 days before necropsy. 14 days was chosen as the infection period in order to quantify larval establishment before the onset of self-cure of larvae from the intestine [32], as well as being the judged optimal for analysis of mucosal immune reactions, due to the transient nature of responses such as eosinophilia [21].

**Fig 2:**
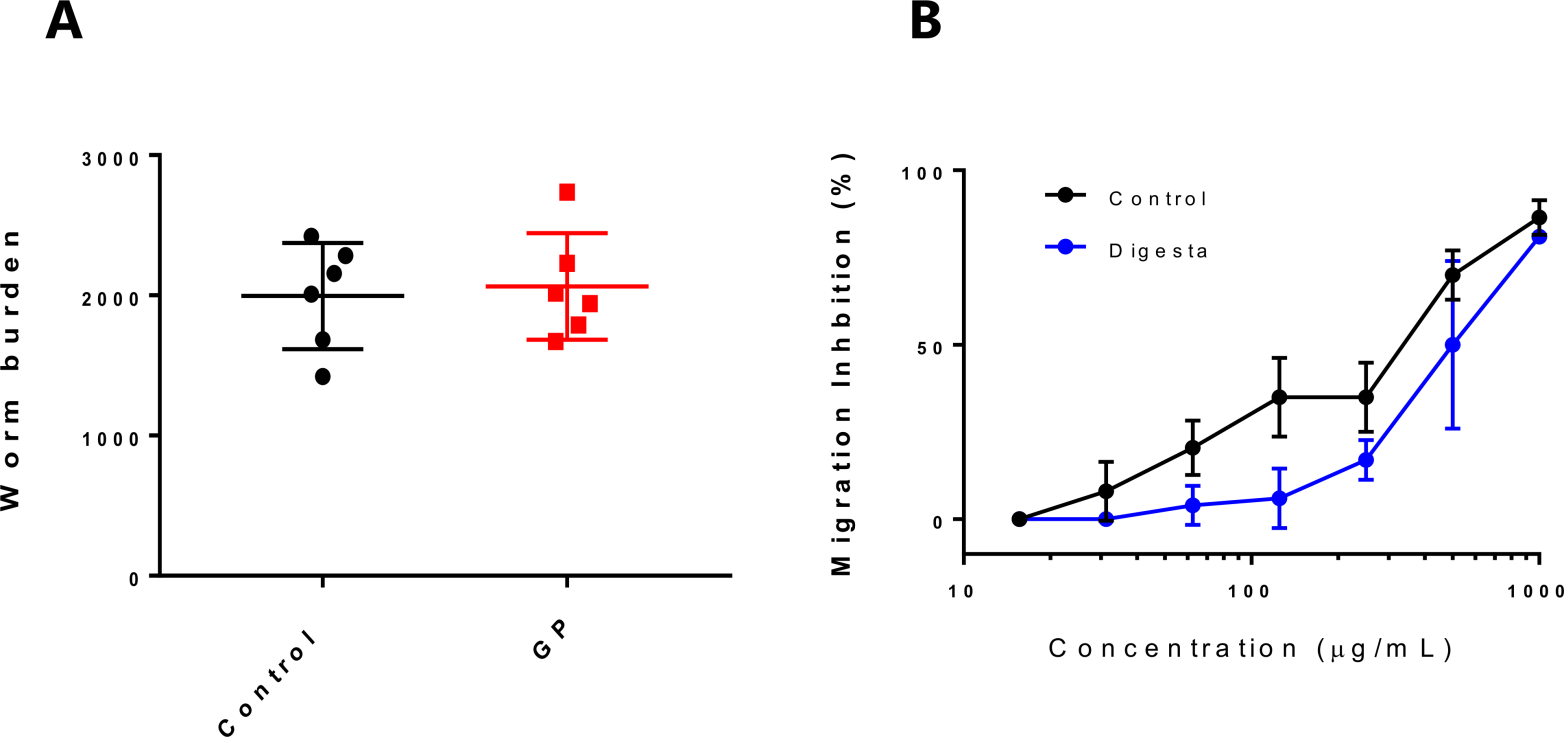
Lack of direct *in vivo* anthelmintic activity of grape pomace (GP) against *Ascaris suum* larvae. **A)** *A*. *suum* burdens (mean ± SD) at day 14 post-infection in pigs fed either a control diet or GP-supplemented diet. **B)** Inhibition of migration of *Ascaris suum* larvae after 24 hours incubation in acetone-water extract of GP, or the same extract pre-incubated with jejunal digesta from pigs.

All pigs were clinically normal during the experiment. No diarrhoea was observed and weight gain did not differ between groups. At day 14 post-infection (p.i.), establishment of *A*. *suum* was not reduced (Fig 2A). Based on our *in vitro* data, we expected that a sufficient concentration of PAC should have been achieved in the intestine to exert some anthelmintic activity. However, polyphenols also bind readily to host and dietary molecules, and so we postulated that non-specific binding to the host intestinal digesta may have prevented sufficient contact between the active components in GP and the larvae. To test this hypothesis, we repeated the *in vitro* larval migration inhibition assay with GP-extracts pre-incubated with jejunal digesta from pigs fed an identical control diet to that used here, and observed a significant increase in the EC_50_ value compared to control extract (504 and 271 μg/mL, respectively, *P*<0.005). Therefore, inclusion of GP in the diet of pigs under these experimental conditions does not appear to have direct anthelmintic activity against *A*. *suum* larvae in the small intestine.

### Effect of dietary grape pomace on parasite-specific antibody responses

**Fig 3:**
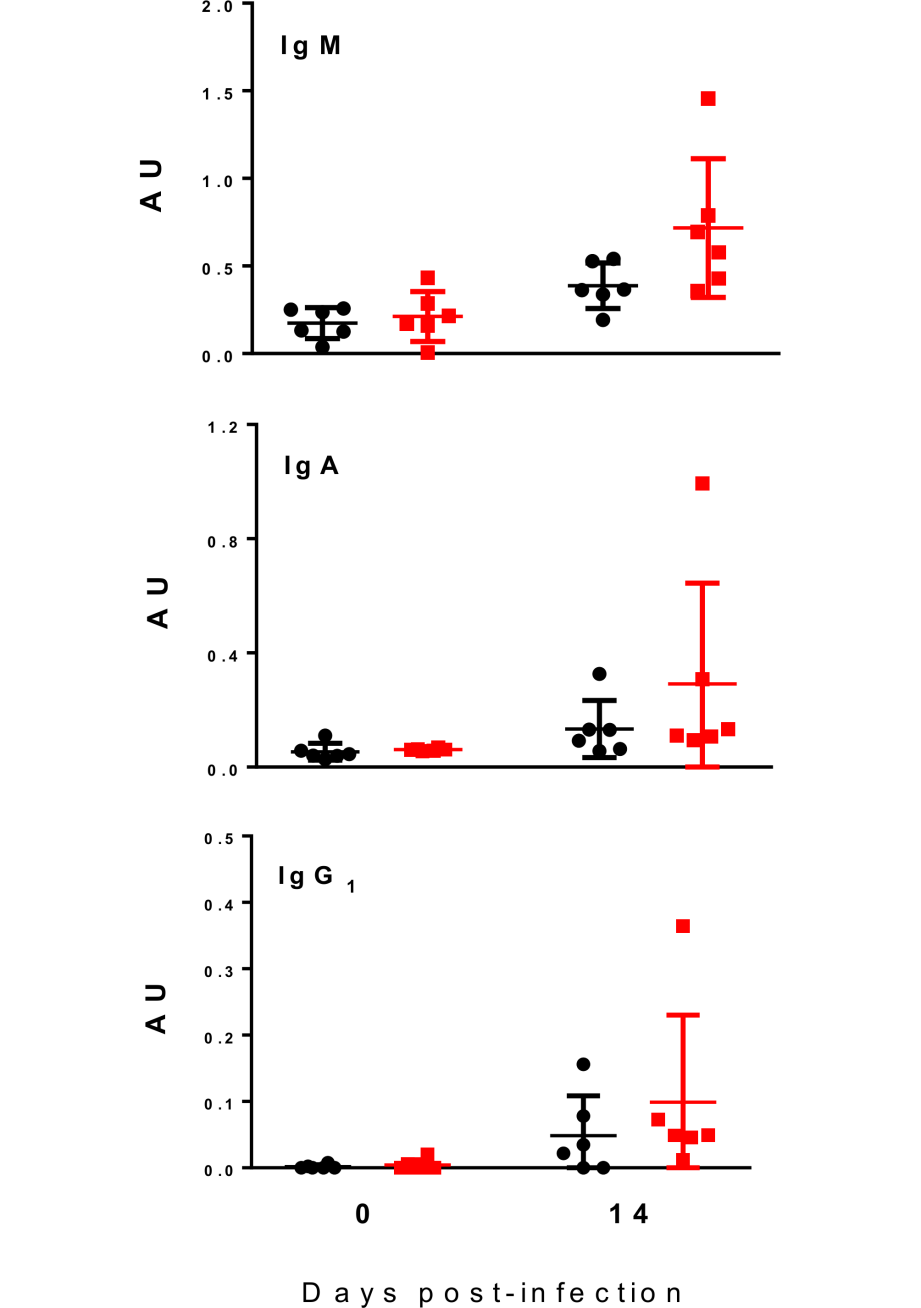
Acquisition of antibodies in *Ascaris suum*-infected pigs fed grape pomace. Levels of plasma IgM, IgA, and IgG_1_ (means ± SD) specific for *A*. *suum*. Values are given for pre-infection samples (Day 0) and for 14 days post-infection in pigs fed either a control diet (black) or a diet supplemented with grape pomace (‘GP’—red). For each antibody isotype values are expressed as a absorbance units (AU). For each group n = 6.

We next assessed whether dietary GP may enhance acquisition of parasite-specific immune function, which may be important for long-term protective immunity and resistance to re-infection. We first assessed the acquisition of parasite-specific antibodies in plasma. Anti-*A*. *suum* IgM, IgA and IgG_1_ levels in plasma were significantly increased at day 14 p.i., and in the case of IgM, there was a tendency (*P* = 0.06) for levels to be higher in infected pigs fed the GP-enriched diet (Fig 3). IgA and IgG_1_ levels were also numerically higher in the pigs fed GP but these differences were not significant.

### Dietary grape pomace regulates mucosal immune responses during *Ascaris* infection

**Fig 4:**
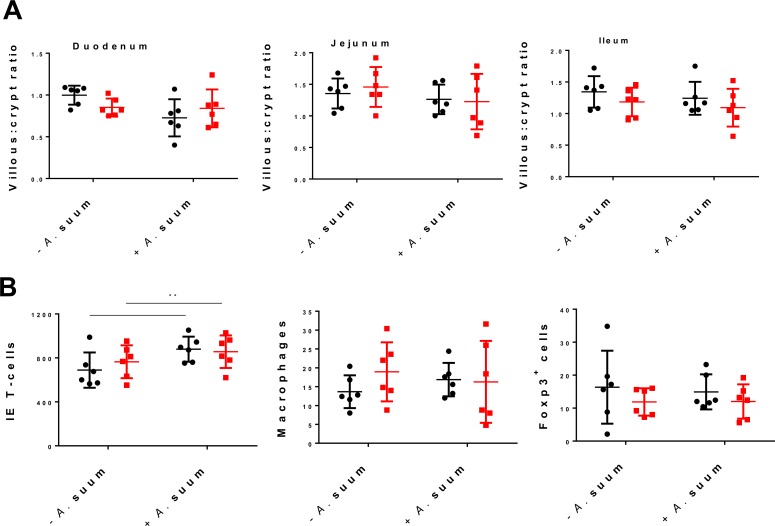
Effect of *Ascaris suum* infection and grape pomace supplementation on mucosal architecture and immune cells in jejunum. Villous: crypt ratios in the duodenum, jejunum and ileum **(A)** and numbers of intra-epithelial T-cells, macrophages and Foxp3^+^ cells in the jejunum **(B)** of pigs fed either a control diet (black) or a diet supplemented with grape pomace (red), and either infected for 14 days with *Ascaris suum* or not infected. Shown is mean ± SD ** *P*<0.01 by two-way ANOVA with Bonferroni post-hoc testing. All cell counts are expressed in cells / mm^2^ tissue.

**Fig 5:**
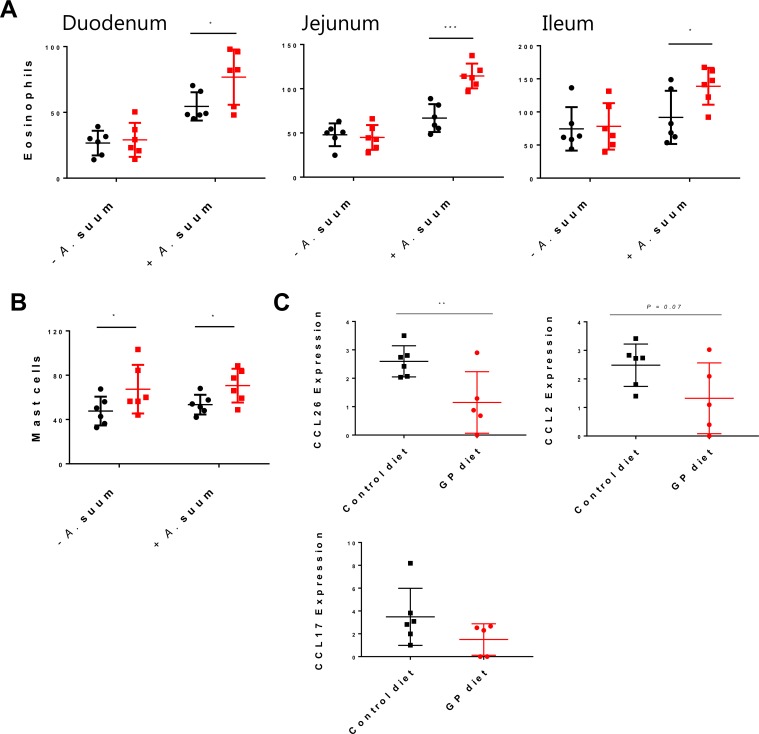
Effect of *Ascaris suum* infection and grape pomace supplementation on mucosal granulocyte numbers and chemokine expression. Numbers of eosinophils in the duodenum, jejunum and ileum **(A)**, mast cells in the jejunum **(B)** and gene expression of three different chemokines in the jejunal mucosa **(C)** of pigs fed either a control diet (black) or a diet supplemented with grape pomace (red), and either infected for 14 days with *Ascaris suum* or not infected. Shown is mean ± SD ***P<0,005, *P<0.05 by two-way ANOVA with Bonferroni post-hoc testing. Gene expression data is relative expression of each gene normalized to the reference genes *B2M*, *RPL13A* and *TBP*. All cell counts are expressed in cells / mm^2^ tissue.

*A*. *suum* is known to induce a strong cellular reaction including increases in intra-epithelial T-cells and mucosal eosinophils in its infection site in the small intestine around 10–14 days p.i., preceding the induction of self-cure mechanisms [21]. Therefore, we next assessed the effect of dietary GP on local cellular responses to *A*. *suum* larvae and mucosal architecture in the small intestine. Villous: crypt ratios (VCR) are often decreased in helminth infection due to increased epithelial cell turnover, and we noted here that VCR tended to be decreased by *A*. *suum* infection in the duodenum (*P* = 0.06), however there were no differences in the jejunum or ileum, and diet did not affect the ratios in any gut segment (Fig 4A). Intra-epithelial T-cells in the jejunum were increased by infection (*P*<0.01), but were not affected by diet (Fig 4B). There was no effect of either diet or infection on macrophages or Foxp3^+^ T-cells in the jejunum (Fig 4B). Mast cell numbers in the jejunum were not increased by infection, but were increased (*P*<0.05) by dietary GP (Fig 5B). Strikingly, eosinophil numbers were strongly affected by diet in *A*. *suum* infected pigs. In all three gut segments measured (duodenum, jejunum and ileum), eosinophils numbers were increased by infection (*P*<0.05) and were significantly higher (*P*<0.05) in infected pigs fed the GP-enriched diet than infected pigs fed the basal diet (Fig 5A). Eosinophil numbers were not increased in uninfected pigs supplemented with GP, indicating that GP synergised with *A*. *suum* infection to regulate eosinophil numbers. To further investigate the role of dietary GP in mucosal eosinophilia during *A*. *suum* infection, local expression levels of genes encoding chemokines involved specifically in granulocyte chemotaxis and function were investigated in *A*. *suum*-infected pigs. Interestingly, there was a consistent trend for the expression of chemokines to be down-regulated by GP-supplementation in infected pigs. *CCL26* expression was significantly down-regulated (*P*<0.01), whilst expression of *CCL2* tended to be down-regulated (*P* = 0.07; Fig 5C). Expression of *CCL17* followed a similar pattern of regulation (negative fold change of 1.8) however, this was not significant. There was no effect of diet on the expression of the Th2/eosinophil-related *IL5* or *IL5RA* in *A*. *suum*-infected pigs, nor were there any changes in the expression of the Th1-type cytokines *IFNG* or *IL8* (S2 Fig).

### Colonic short-chain fatty acid (SCFA) production is regulated by dietary grape pomace and *Ascaris* infection

Helminths are known to impact upon gastrointestinal function and microbiota composition, due to both their direct physiological as well as immunomodulatory effects on the host. To investigate whether *A*. *suum* infection and/or GP-supplementation induced changes in gut function, we first assessed SCFA concentrations in faecal samples taken from the distal colon. Independently of infection status, GP-supplementation increased the concentration of propionic acid (*P*<0.005; Table 2), a known modulator of inflammation and regulatory immune responses [33]. In contrast, GP-supplementation and *A*. *suum* infection both decreased the concentrations of isobutyric acid and isovaleric acid (*P*<0.05), however there was not an additive effect of diet and infection (Table 2). There was no effect of either diet or infection on concentrations of acetic, butyric or valeric acid.

**Table 2:** Short chain fatty acids concentration (SCFA; mmol/kg wet sample) in the distal colon of pigs infected or not with *Ascaris suum*, and either fed a control diet or a diet supplemented with 5% grape pomace (GP). Each group n = 6. SCFA in bold and italicised are significantly impacted by infection and/or diet.

	*- A*. *suum*		+ *A*. *suum*		*P* value		
Diet	Control	GP	Control	GP	Infection	Diet	Interaction
Acetic acid	57.4 ± 1.6	59.9 ± 1.83	55.4 ± 1.3	55.7 ± 3.1	0.18	0.54	0.64
***Propionic acid***	***22*.*5 ± 0*.*8*** ^***A***^	***25*.*6 ± 0*.*7*** ^***B***^	***20*.*6 ± 1*.*9*** ^***A***^	***24*.*8 ± 1*.*1*** ^***B***^	***0*.*18***	***0*.*002***	***0*.*56***
Butyric acid	14.5 ± 1.6	14 ± 1	13.1 ± 1.2	13.2 ± 1	0.36	0.87	0.81
Valeric acid	4.3 ± 0.3	3.9 ± 0.4	3.7 ± 0.6	3.8 ± 0.4	0.47	0.67	0.62
***Iso-butyric acid***	***2*.*7 ± 0*.*4*** ^***A***^	***1*.*4 ± 0*.*1*** ^***B***^	***1*.*6 ± 0*.*1*** ^***B***^	***1*.*7 ± 0*.*2*** ^***B***^	***0*.*14***	***0*.*026***	***0*.*015***
***Iso-valeric acid***	***1*.*9 ± 0*.*2*** ^***A***^	***0*.*9 ± 0*.*1*** ^***B***^	***1 ± 0*.*1*** ^***B***^	***1*.*1 ± 0*.*1*** ^***B***^	***0*.*13***	***0*.*023***	***0*.*014***

^A,B^ Values followed by different subscripts differ (P*<*0.05) by 2-way ANOVA with Bonferroni post-hoc testing.

### Gut microbiota composition is modified by both *Ascaris* infection and dietary grape pomace

**Fig 6:**
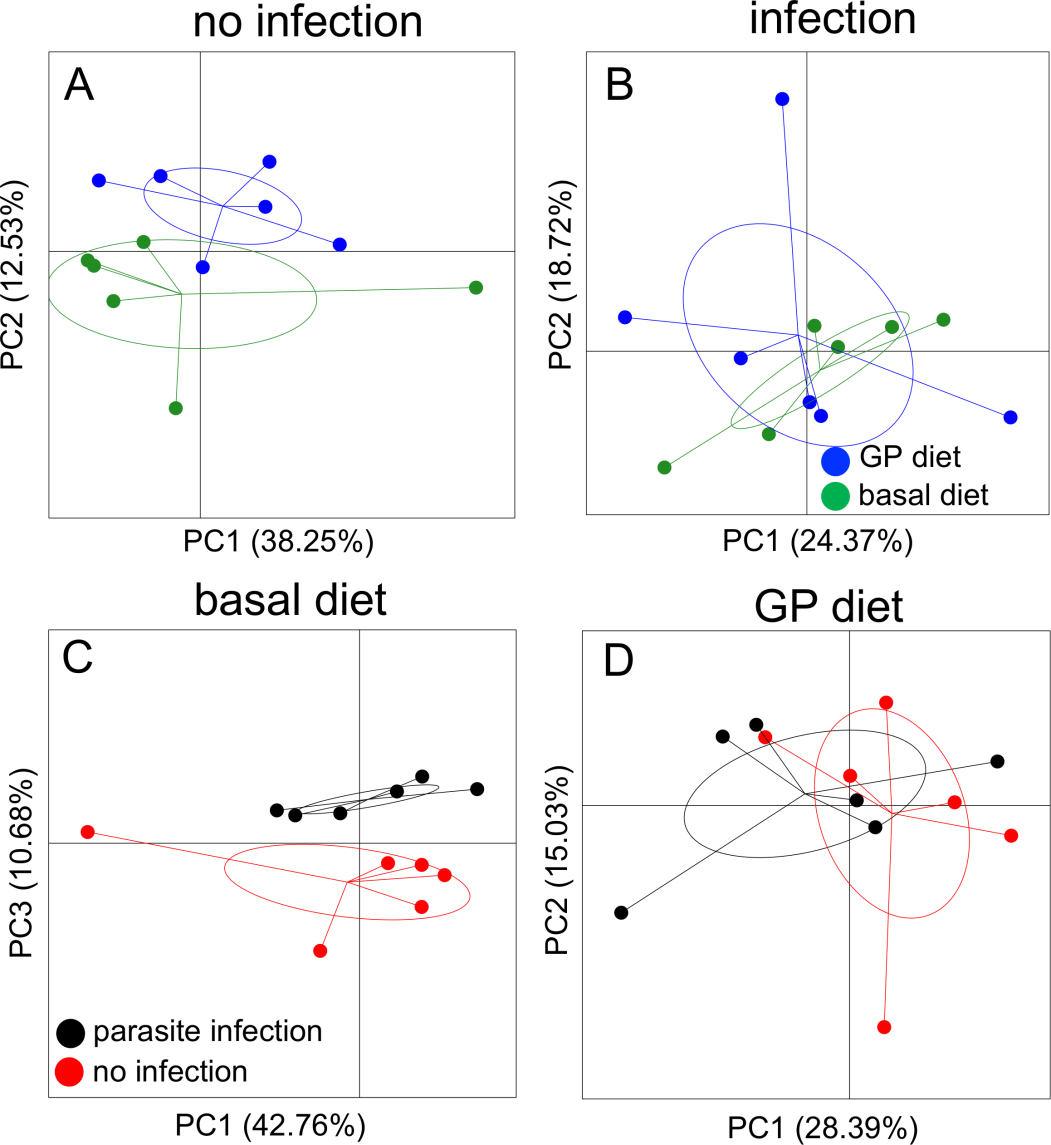
Effect of *Ascaris suum* infection and grape pomace supplementation on gut microbiota composition. Gut microbiota (GM) composition was determined by tag-encoded 16S rRNA gene amplicon sequencing. Principal Coordinates Analysis (PCoA) plots based on generalized UniFrac distance matrices showing: (A) the effect of diet on GM composition of non-infected pigs (*P<*0.05 by MANOVA); (B) lack of the diet effect on GM composition of *A*. *suum*-infected pigs (*P =* 0.798 by MANOVA,); (C) the effect of *A*. *suum* infection on the GM composition of pigs fed the basal diet (*P*<0.01 by MANOVA); (D) lack of the infection effect on GM composition of pigs feed with the GP-supplemented diet (*P =* 0.437 by MANOVA).

To explore if *A*. *suum* and/or diet induced changes in the host prokaryotic gut microbiota, digesta samples from the proximal colon were analysed by tag-encoded 16S rRNA-based amplicon sequencing. Principal Coordinate Analysis (PCoA) of generalized UniFrac distance metrics showed that the effect of diet on the gut microbiota composition was clear among non-infected pigs (Fig 6A; *P* < 0.05 by MANOVA on two respective PCs). In contrary, no effect of diet on the microbiota composition was seen amongst the infected pigs (Fig 6B; *P =* 0.798 by MANOVA on two respective PCs). Similarly, *A*. *suum* infection altered the microbiota in pigs fed the basal diet (Fig 6C; *P* < 0.001 by MANOVA on two respective PCs). However, this effect was suppressed among pigs fed the GP-enriched diet (Fig 6D; *P =* 0.437 by MANOVA on two respective PCs), indicating a marked interaction between diet and infection on the composition of the microbiota. The alpha diversity analysis showed a trend for increased microbial diversity of *A*. *suum* infected pigs fed the basal diet compared to non-infected pigs feed the same diet (*P* = 0.065; S3A Fig). No differences in diversity were seen between infected and non-infected pigs fed with the GP-enriched diet (S3B Fig).

The dominant genus in all pigs was *Prevotella*, accounting for 30% of bacterial population in the control animals. The proportion of *Prevotella* was 45 and 50% in the other three groups, however these differences were not significant (Table 3). A total of 13 genera were significantly impacted by infection (Table 3), with notable increases in OTUs closely related to *Facklamia* and *Turicibacter*, and a decrease in *Ruminicoccus* and *Lactobacillus*. Similar to *A*. *suum* infection, the relative abundance of *Lactobacillus* and *Ruminicoccus* were lower in pigs fed the GP-enriched diet, whilst there was an increase in *Treponema* and *Campylobacter* (Table 3).

**Table 3:** Bacterial genus operational taxonomic units (OTUs) in the proximal colon of pigs infected or not with *Ascaris suum*, and either fed a control diet or a diet supplemented with 5% grape pomace (GP). For each group N = 6. Shown is the most abundant genus (*Prevotella*) and genera that were impacted by either infection or diet.

	Control		*A*.*suum-*Infected		*P* value		
Genus (closest related)	Basal diet	GP diet	Basal diet	GP diet	Infection	Diet	Interaction
*Campylobacter*	349	1105	120	610	0.21	0.03	0.64
*Catenibacterium*	11. 7^A^	4.2^A^	3.5^B^	4.5^A^	0.034	0.073	0.022
*Cellulosimicrobium*	0	0	0.33	0.5	0.039	0.84	0.84
*Facklamia*	0.2	0.5	4.5	3	0.006	0.72	0.52
*Haemophilus*	0.3	0.2	1.5	2.3	0.049	0.78	0.68
*Lactobacillus*	1258.2^A^	407.8^A^	317.8^B^	521.1^A^	0.12	0.18	0.045
*Prevotella*	6423.2	9292.2	9032.8	9110.2	0.45	0.34	0.2
*Ruminicoccus*	107.7	47.8	60	53.6	0.15	0.03	0.055
*Succinivibrio*	37	36.8	116.7	71.8	0.048	0.39	0.42
*Treponema*	2.5	8.7	4	6.5	0.95	0.046	0.29
*Turicibacter*	1.7	1.3	6.3	10	<0.0001	0.35	0.25
Unclassified *Campylobacteraceae*	4.2	9.5	2.2	9.2	0.66	0.03	0.75
Unclassified *Paraprevotellaceae*	4.8	5.7	17.8	12.5	0.022	0.23	0.19
Unclassified *Pasteurellaceae*	0.3	0	3	4.5	0.02	0.88	0.38
Unclassified *Planococcaceae*	0.3	0.2	4.7	1.5	0.011	0.25	0.48
Unclassified *Porphyromonadaceae*	1.3	1.3	2.5	5	0.048	0.31	0.31
Unclassified *Succinivibrionaceae*	2	3.3	9	6.2	0.029	0.73	0.33
Unclassified *Veillonellaceae*	229.8	218.3	114.7	97.3	0.033	0.78	0.95

^A,B^ Within genera, values followed by different subscripts differ (P*<*0.05) by 2-way ANOVA followed by Bonferroni post-hoc testing.

## Discussion

Bioactive dietary components such as polyphenols have received a great deal of interest in recent times for their apparent ability to reduce infection and/or egg shedding of pathogenic parasites such as *Haemonchus contortus* and *Ostertagia ostertagi* [11, 34], as well as enhancing immune function [35]. In our current study, we found that whilst establishment of *A*. *suum* was not impacted by dietary GP, modulation in the initiation of cellular immune responses to *A*. *suum* infection were observed.

A previous study with growing pigs has shown that inclusion of grape meal extract (polyphenol content of 8.5%) at 1% of dietary intake was sufficient to induce changes in gut bacterial populations and inflammatory gene expression [31]. Here, we chose a higher inclusion level of 5% GP (equating to a daily intake of around 6–8 g of PAC) in order to increase possible anthelmintic activity. This was extrapolated from concentrations showing activity in our *in vitro* experiments, as well as pilot experiments that demonstrated that pigs readily consumed this amount of GP in the diet. Whilst dose-response experiments were outside the scope of this study, there are reports that higher amounts of GP (≥10%) may depress feed intake and growth, which suggests an upper limit of GP that can be incorporated into pig diets [36]. However, further investigations with higher doses of purified PAC may be useful to determine if such an approach can mediate direct anthelmintic effects against *A*. *suum* larvae, and may also be relevant as a 'functional food' concept in humans. We did not perform characterisation and quantification of PAC in the jejunum, so we cannot confirm whether local concentrations of PAC were too low due to absorption or metabolism. However, absorption of PAC in the small intestine of monogastric animals is thought to be minimal [37], and our experiments suggest that the discrepancy between our *in vitro* and *in vivo* anthelmintic data may be related to non-specific binding of polyphenols in GP to endogenous host or digesta molecules,. Whilst this may reduce the ability of GP to mediate direct anthelmintic effects, the ability of polyphenols to interact with host cells is speculated to impart their putative immunomodulatory and anti-inflammatory properties [30, 38].

Consequently, whilst this level of dietary GP did not appear to be sufficient to directly reduce establishment of *A*. *suum* in this infection model, the strong mucosal immune responses induced at this early time-point (day 14 p.i.) allowed us the opportunity to examine in detail the effect of GP-supplementation on the acquisition of parasite-specific immune responses. Whilst specific antibody levels in plasma were not significantly increased by diet, there was a possible trend for all three isotypes (IgM, IgA and IgG_1_) to be increased by GP-supplementation. This is in agreement with previous work in sheep showing increases in helminth-specific antibody titres in animals fed PAC-rich forages, despite a clear lack of reductions in parasite burdens [35, 39]. Furthermore, the increase in mucosal mast cells and eosinophils in pigs fed the GP-enriched diet is also consistent with studies in sheep demonstrating higher granulocyte numbers in parasite-infected animals fed either PAC-rich willow or sainfoin fodders [35, 40]. In these earlier studies with ruminants, these effects on immune function may be related to either direct immunostimulatory effects of bioactive compounds within these fodders, or may be related to improve nutrition, as it is known in ruminants that PAC-containing forages can improve protein supply to the small intestine by binding to and preventing microbial degradation of proteins in the rumen [8]. Our results are the first to demonstrate an increase in granulocytes in a monogastric (i.e. non-ruminant) animal fed a polyphenol-rich diet, and thus provide support for a direct immunological effect of bioactive compounds within GP. Whilst the identify of these compounds cannot be definitively stated, the oligomeric PAC within GP would be prime candidates.

Possible mechanisms of this apparent immunostimulatory effect may include increased activation of mucosal T-cells, especially γδ T-cells for which PAC are strong agonists in a many animal species, including pigs [15, 29], and γδ T-cells have been shown to recruit granulocytes to the mucosa at the site of intestinal infections [41]. Moreover, we have recently demonstrated that human dendritic cells exposed to PAC *in vitro* up-regulate Th2-associated OX40L and subsequently increase IL4/IFNγ ratios in naïve T-cells [30]. Given that mast cells and eosinophils are both dependent on a Th2-environment for their recruitment and survival, whilst Th1-skewing environments inhibit the recruitment of these cells [42], PAC and other polyphenols may directly influence the local immune milieu which results in mast cell and eosinophil recruitment. However, we did not observe a down-regulation of typical Th1-type genes such as *IFNG* in infected pigs fed GP, and further experiments are clearly necessary to unravel these mechanism(s).

It is also interesting to note that gene expression of *CCL26*, which encodes the main chemokine involved in eosinophil chemotaxis, was significantly down-regulated in the jejunal mucosa in *A*. *suum-*infected pigs fed the GP-enriched diet. Why *CCL26* expression should be down-regulated, whilst tissue eosinophilia was increased, is not clear, but it does add further evidence to suggest that dietary GP modulates eosinophil homeostasis in the gut mucosa of infected animals. As local intestinal eosinophilia is normally a transient response in primary *A*. *suum*-infections [21], one hypothesis is that CCL26 production is supressed in GP-fed animals relative to control-fed animals as a homeostatic mechanism in order to counter the stronger influx of cells into the mucosa during the peak eosinophil response. Further time-course experiments investigating the expression of CCL26 before, during and after eosinophil infiltration will be necessary to unravel this relationship. The boosting of the eosinophil-response by dietary GP is notable as it has been observed that eosinophilia is a key mechanism of acquired immunity against *A*. *suum* that eventually results in pre-hepatic immunity following re-infection [27]. Thus, if dietary components can enhance the eosinophil response this may possibly hasten the acquisition of this pre-hepatic barrier. Overall, our results suggest a significant influence of dietary polyphenols on local mucosal responses to *A*. *suum*.

Whilst the total concentrations of SCFA in the colon were not affected by either *A*. *suum* infection or GP-supplementation, both factors selectively regulated the concentrations of individual SCFA. Notably, GP-supplementation enhanced the production of propionic acid, which has been previously linked to reduced inflammation, induction of T-regulatory (Treg) cells and improved colonic health [33, 43]. Of interest, we have previously noted that purified PAC fractions increase production of the regulatory cytokine IL-10 in human DCs, and Park *et al*. [44] noted that administration of PAC-rich grape seed extract to mice increased Tregs. In our current study utilising an acute infection model we did not observe an increase in Foxp3^+^ cells in the jejunum. Despite this, it would be of interest to further determine the effects of GP and other polyphenols on regulatory responses during chronic infections, as induction of Tregs may have implications for the ability of hosts to expel parasites. In contrast, *A*. *suum* infection had no effect on propionic acid concentrations, and neither infection nor diet impacted acetic or butyric acid levels. This is in contrast to Zaiss et al. [45] who reported that *A*. *suum* infection increased butyrate and propionate levels in the colon of pigs with a chronic *A*. *suum* infection. The difference in these observations may relate to the time-point of sampling: in our study, measurements were made at day 14 p.i. at the beginning of the main larval expulsion phase, whilst Zaiss et al. investigated SCFA concentrations at day 56 p.i., during an established adult worm infection. The different stages of the infection (acute compared to chronic) likely result in quite different physiological and immunological states, which may markedly influence parasite-induced changes in gut function. In our current study, we observed that both *A*. *suum-*infection and GP-supplementation both resulted in a significant reduction of the branched-chain SCFA iso-butyric acid and iso-valeric acid, which are normally produced by the fermentation of proteins [46]. This suggests that less protein was available as substrate in the colon. The reasons for this are not clear, but it is known that PAC readily form insoluble complexes with proteins in the digestive tract which pass into the faeces, which may prevent their availability to microbes in the colon [37]. The effect of *A*. *suum* on host protein digestion is not known, but in other animal species (e.g. sheep) helminth infection in the small intestine results in altered protein metabolism including sequestration to the site of infection to replace endogenous secretions and aid in turnover and/or repair of the mucosal epithelium [47]. As *A*. *suum* infection results in reduced net feed efficiency [1], it is also likely that modification of protein metabolism occurs in infected pigs. Overall, both *A*. *suum* infection and GP-supplementation appear to have similar modulatory effects on branched-chain SCFA production in the colon.

*A*. *suum* infection induced a significant change in the composition of the gut microbiota in the proximal colon of pigs fed the basal diet, what is consistent with other studies showing that helminth infection is associated with modulation of the commensal microbiome [19, 20, 48]. Notable changes included an increase in relative abundance of an OTU closely related to *Facklamia*, which has also been shown to be increased by *Opisthorchis viverrini* infection [49]. Moreover, bacterial species diversity tended to be increased by infection, which has also previously been observed in humans infected with soil-transmitted helminths [50, 51]. The functional effects of helminths on the gut microbiota are not yet clear, and may vary markedly according to the parasite species and intensity of infection. Reports range from negative effects, such as repression of carbohydrate and lysine metabolism [19] to potentially positive effects such as maintenance of immunological tolerance and reduced inflammation [45, 51].

The effect of diet on the gut microbiota composition was observed solely in the non-infected group. This is in agreement with other studies on pigs which have showed changes in the microbiota composition following dietary supplementation with GP or related, polyphenol- and PAC-rich additives [16, 31]. Interestingly, the effect of diet was not evident in the *A*.*suum* infected group, and, conversely, the effect of infection was not significant in pigs fed the GP-enriched diet. Therefore, it appears that infection and GP-supplementation may drive the microbiota compositional changes in similar directions, with the result of masking the effect of diet in the infected group, and the effect of infection in the GP-supplemented group. Examination of bacterial genera that were significantly altered suggested some comparable trends of diet and infection, particularly the reduction of *Lactobacillus*, *Ruminococcus* and *Catenibacterium* by both *A*. *suum* and GP-supplementation. Perhaps unexpectedly, pigs fed the GP-enriched diet had a higher abundance of *Campylobacter*. Interestingly, *Campylobacter* has also been reported to be increased in pigs infected with *Trichuris suis*, perhaps arising due to the modulation of the Th2/Th1 balance in infected animals [52]. The overlapping effects of *A*. *suum*-infection and diet on the microbiota appear to be consistent with the similar effects on branched-chain SCFA production and mucosal eosinophilia. Whether these observations are mechanistically linked is unknown, but given that there appears to be strong inter-relationships between helminth induced-changes in the gut microbiota and establishment of Th2-type immunity [53], possible links between diet and parasites on gut microbiota-mediated immunological changes should be investigated. In addition, it may also be of interest to determine whether there exist differences between sexes in the response to GP-supplementation, as our study used castrates rather than entire males, and sex may influence diet-induced changes in microbiota composition and immune function [54].

In conclusion, our study demonstrates that supplementation of pig diets with polyphenol-rich GP, an increasingly popular feed additive in swine production, increased mucosal eosinophil and mast cell numbers during *A*. *suum* infection. Both *A*. *suum* and dietary GP also appeared to modulate gut function by regulating prokaryotic gut microbiota composition and/or changing SCFA concentrations in the colon. Further studies should focus on definitive isolation and identification of the bioactive molecules within GP, as well as improving delivery of concentrated doses of polyphenols. Moreover, whether there are long-term benefits of bioactive diets on gut health and immunity during parasite infection should be addressed, as well as the underlying mechanisms underpinning this novel interaction between diet and immune function.

## Materials and methods

### Extraction and analysis of proanthocyanidins in grape pomace

For *in vitro* assays, extracts were prepared from the GP using acetone-water extraction followed by evaporation and freeze-drying [6]. Total PAC content of raw GP (Nor-Feed A/S, Hvidore, Denmark) and GP acetone-water extracts were determined using the HCl-butanol method [55]. The PAC were further subjected to thiolytic degradation and HPLC-MS analysis, essentially as described [6], in order to determine the mean degree of polymerization (mDP), proportion of monomeric flavan-3-ol units and the percentage of galloyation.

### Anthelmintic effects of grape pomace extract *in vitro*

*In vitro* anthelmintic activity against *A*. *suum* was assessed using motility scoring and an agar-based larval migration inhibition assay as previously described [6, 28]. Briefly, embryonated *A*. *suum* eggs were hatched by stirring with glass beads at 37°C for 30 minutes, and the viable third-stage larvae (L3) were purified by overnight migration in a Baermann apparatus. Larvae were then exposed for 24–72 hours to the GP extract (dissolved in culture medium–RPMI1640 medium with 2 mM L-glutamine, 100 U/mL penicillin and 100 μg/mL streptomycin) or to medium only. Motility scoring was done based on a 0–5 scale where 5 is fully motile and 0 is completely immotile. Levamisole (25 μg/mL) was included as a positive control for inhibition of motility. For the migration assay, after 24 hours incubation larvae were incorporated into 0.5% agar. After an overnight migration period, the number of migrating larvae was counted and the inhibition of larval migration relative to the medium-only control was calculated. To test the effect of intestinal content on the anthelmintic activity of the GP extract, the extract was serially diluted from 1000 to 7.5 μg/mL. Intestinal content was obtained from the jejunum of pigs fed the same basal control diet as used in the *in vivo* trial and stored at -80°C. Each concentration of extract was then incubated with an equal amount of digesta for 4 hours at 37°C. Controls consisted of digesta incubated with medium alone, or extracts or media containing no digesta that were similarly incubated. The extracts with or without digesta were then centrifuged briefly, and the supernatant filtered through a 0.2μm syringe filter and then used for the migration assay. EC_50_ values were calculated by non-linear regression and differences in EC_50_ and regression slope calculated by extra-sum-of-squares F-test, as described previously [6].

### Animals and diets

24 helminth-naïve pigs (12 females and 12 castrated males, Danish Landrace-Yorkshire, aged 7–8 weeks with a mean weight of 21 kg on arrival) were obtained from a specific pathogen-free farm with no history of helminth infection (Frenderupgaard, Sorø, Denmark). The pigs were confirmed copro-negative and sero-negative for *A*. *suum* infection on arrival. The animals were stratified on the basis of sex and weight and randomly allocated to 4 groups (each n = 6). Two groups were fed a basal diet consisting of a commercial grower pig diet based on ground barley and soybean meal consisting of 16.4% crude protein (NAG, Denmark). The other two groups were fed the basal diet containing 5% GP (w/w) that was thoroughly mixed into the diet prior to each meal. The amount of feed offered to the different groups each day was calculated to ensure an equal daily intake of crude protein and metabolizable energy. Animals were observed twice daily by experienced personnel for clinical signs of helminth infection or intestinal disease such as diarrhoea, inappetence, depression or poor growth. All pigs were weighed weekly.

### Parasite infection and post-mortem procedures

After 7 days of acclimatisation to the diets, 6 pigs on the basal diet and 6 pigs on the GP-enriched diet were inoculated by gavage with 5000 embryonated *A*. *suum* eggs. 14 days post-infection, the animals were killed by captive bolt pistol followed by exsanguination. Blood was collected into EDTA-coated vacutainers by venepuncture of the jugular vein prior to infection and immediately prior to slaughter. Plasma was separated by centrifugation and stored at -20°C. Full-thickness tissue samples were taken from the duodenum and jejunum (10 cm and 4.5 m distal to the pylorus, respectively) and the ileum (20 cm proximal to the ileao-caecal junction), washed with ice-cold PBS and sections (1 cm^2^) were placed into 4% formaldehyde. An additional section from the jejunum was placed into Carnoy’s fixative. Mucosal scrapings were also taken from the same jejunal site by scraping the mucosa with a glass slide, and the samples snap-frozen in liquid nitrogen and stored at -80°C. Digesta samples were also taken from the proximal colon (15 cm distal to the ileal-caecal junction) and distal colon (75cm distal to the anus), snap-frozen in liquid nitrogen and stored at -80°C.

### Histology and immunohistochemistry

Paraformaldehyde-fixed sections were paraffin embedded, sectioned and stained with haematoxylin and eosin for enumeration of eosinophils and villus: crypt ratios. An additional section from the jejunum was processed for immunohistochemical determination of T-cells, macrophages and Foxp3^+^ cells using the follow antibodies, respectively: rabbit anti-human CD3 (Dako), mouse anti-human macrophage (clone MAC387, Abcam) and mouse anti-human Foxp3 (clone 22510, Abcam). Appropriate isotype controls were used to determine antibody specificity. Bound antibodies were visualised with the Ultravision LP Detection System and aminoethyl carbazole substrate (ThermoFisher Scientific, Denmark). The Carnoys-fixed jejunum section was stained with Toluidine blue for mast cell enumeration. For determination of cell numbers, 10 random fields of each tissue section were counted by a single blinded observer using a calibrated counting grid covering a total area of 0.25mm^2^ at 400x magnification. Cell counts were expressed as cells/mm^2^ tissue. For villous: crypt ratios, 10 well-orientated villus: crypt units were randomly selected from each tissue section by a blinded observer. The sections were photographed using a Leica DFC480 camera and measurements performed using LAS v4.6 software (Leica, Switzerland).

### ELISA

Anti-*A*. *suum* IgM, IgA and IgG_1_ levels in plasma were detected by ELISA using adult body fluid (ABF) derived from adult worms as antigen. The ABF preparation and ELISA was conducted as described previously [56]. Briefly, plates were coated with ABF (5 μg/mL) overnight at 4°C and specific antibodies in plasma were detected by the addition of goat anti-pig IgM or IgA-horseradish peroxidase (HRP) conjugate, or mouse anti-pig IgG1 (clone K139 3C8) followed by rat anti-mouse IgG-HRP (all from Abd Serotec). On every plate, a standard curve was constructed using hyper-immune plasma samples. Sample dilutions that fell within the linear portion of the standard curve were used to determine the sample values, and expressed as absorbance units.

### Gene expression analysis

RNA was extracted from jejunum mucosal scrapings using the miRNAeasy kit (Qiagen) including on-column DNAase treatment. Quality of the extracted RNA was assessed using the Aligent 2100 Bioanalyzer system. First-strand cDNA synthesis, including gDNA removal, was performed using the QuantiTect Reverse Transcription Kit (Qiagen) according to the manufacturer’s instructions. Three cDNA replicates were synthesized for each RNA sample. qPCR was performed using 48.48 dynamic arrays (Fluidigm, CA) using a as described previously [56]. Briefly, for each sample we prepared a sample mix consisting of 1.5 μL pre-amplified cDNA (diluted 1:10 in low-EDTA TE buffer (VWR-Bie & Berntsen, Denmark)), 3 μL of 2X TaqMan Gene Expression Master Mix (Applied Biosystems), 0.3 μL 20X DNA binding dye (Fluidigm), 0.3 μL 20X EvaGreen (Biotium, VWR-Bie & Bernsten) and 0.9 μL low-EDTA TE buffer. Next, for each primer pair a primer mix was prepared consisting of 2.5 μL 2X Assay loading reagent (Fluidigm), 0.25 μL low-EDTA TE buffer and 2.3 μL of 20 μM forward and reverse primer. qPCR was performed on a BioMark HD Reader (Fluidigm) using the following conditions: 2 minutes at 50°C, 10 minutes at 95 °C followed by 35 cycles of 15 seconds at 95°C and 1 minute at 60°C. Melting curves were generated by increasing the temperature from 60°C to 95°C after end PCR. For normalization, experimental data was normalized against a mean of the three reference genes *B2M*, *RPL13A* and *TBP*, which were selected using NormFinder [57] and GeNorm [58]. Triplicate cDNA samples were averaged after normalisation, and log_2_ transformed prior to statistical analysis. Primer sequences are listed in S1 Table.

### Measurement of colonic short-chain fatty acid (SCFA) production

Distal colon contents were extracted and the SCFA concentrations determined by gas chromatography as previously described [59].

### Gut microbiota analysis

The proximal colon gut microbiota composition was determined using tag-encoded 16S rRNA gene MiSeq-based (Illumina, CA) high throughput sequencing. Cellular DNA was extracted using PowerSoil DNA Isolation Kit (Mo Bio Laboratories, CA) according to the manufacturer’s instructions including an initial bead-beating step using FastPrep-24 5G (MP Biomedicals, CA). The V3 region (approximately 190 bp) of the 16S rRNA gene amplified using primers compatible with Nextera Index Kit (Illumina; NXt_388_F: 5′–TCGTCGGCAGCGTC AGATGTGTATAAGAGACAG ACWCCTACGGGWGGCAGCAG–3′ and NXt_518_R: 5′–GTCTCGTGGGCTCGG AGATGTGTATAAGAGACAG ATTACCGCGGCTGCTGG–3′). Polymerase chain reactions (PCR) containing 12 μL AccuPrime SuperMix II (Life Technologies, CA), 0.5 μL of each primer (10 μ*M*), 5 μL of genomic DNA (approximately 20 ng/μL), and nuclease-free water to a total volume of 20 μL were run on a SureCycler 8800 (Agilent, CA). Standard PCR cycling was performed with an initial denaturation at 95°C for 2 min and followed by 33 cycles of 95°C for 15 sec, 55°C for 15 sec, and 68°C for 40 sec, and a final elongation at 68°C for 5 min. PCR products from the first PCR step were used as templates in second PCR step by incorporating specific primers with adapters and indexes. 2 μL of PCR product were used in the second PCR reactions containing 12 μL Phusion High-Fidelity PCR Master Mix (Thermo Fisher Scientific, MA), 2 μL corresponding P5 and P7 primer (Nextera Index Kit), and nuclease-free water for a total volume of 25 μL. Cycling conditions applied were 98°C for 1 min; 12 cycles of 98°C for 10 sec, 55°C for 20 sec, and 72°C for 20 sec; and elongation at 72°C for 5 min. The amplified fragments with adapters and tags were purified using size-exclusion AMPure XP beads (Beckman Coulter Genomic, CA). Prior to library pooling, clean constructs were quantified using a Qubit fluorometer (Invitrogen, Carlsbad, CA) and mixed in approximately equimolar concentrations to ensure even representation of reads per sample followed 250 bp pair-ended MiSeq (Illumina) sequencing performed according to the instructions of the manufacturer.

The raw data set containing pair-ended reads with corresponding quality scores were merged and trimmed using fastq_mergepairs and fastq_filter scripts implemented in the UPARSE pipeline. The minimum overlap length of trimmed reads (150 bp) was set to 100 bp. The minimum length of merged reads was 150 bp. The maximum expected error E = 2.0, and first truncating position with quality score N ≤ 4. Purging the data set from chimeric reads and constructing de novo operational taxonomic units (OTU) were conducted using the UPARSE pipeline (uchime_ref). The green genes (13.8) 16S rRNA gene collection was used as a reference database. The Quantitative Insight Into Microbial Ecology (QIIME) open source software package (1.7.0 and 1.8.0) was used for subsequent analysis steps [60].

### Data analysis and statistics

For analysis of microbiota composition and diversity, principal coordinate analysis (PCoA) was conducted with the Jackknifed Beta Diversity workflow based on 10 distance metrics calculated using 10 subsampled OTU tables and plotted with R. The number of sequences taken for each Jackknifed subset was set to 85% of the sequence number within the most indigent sample (20,000 reads/sample). Multivariate Analysis of Variance (MANOVA) was used to evaluate group clustering along first three PCoA components. MANOVA was chosen over ‘Adonis’ (R, ‘vegan’) due to the limited samples number. Alpha diversity measures expressed with an observed species (sequence similarity 97% OTU) value were computed for rarefied OTU tables (20,000 reads/sample) using the alpha rarefaction workflow. Differences in alpha diversity were determined using a *t*-test–based approach using the nonparametric (Monte Carlo) method (999 permutations) implemented in the compare alpha diversity workflow.

The main effects and interaction of diet and infection on mucosal histology and cell numbers, plasma antibody levels, SCFA concentrations and bacterial genera OTU were assessed by two-way ANOVA followed by Bonferroni post-hoc testing. All data was checked for normality by Shapiro-Wilk testing. Bacterial OTU abundances were square-root transformed prior to analysis. The effect of diet on gene expression in infected pigs was assessed by students t-test on log_2_-transformed data, and reported as relative expression. Analyses were performed using Graphpad Prism v6.0, and significance was taken at *P*<0.05, with *P*<0.1 also reported as trends.

## Supporting information

S1 Table Primers sequences and amplicon lengths used in gene expression analysis. (DOCX)

S1 Fig Structures of flavan-3-ol monomeric subunits and an example of a tetrameric proanthocyanidin (condensed tannin). (DOCX)

S2 Fig Gene expression of *IL5*, *IL5RA*, *IL8* and *IFNG* in the jejunal mucosa of *A*. *suum*-infected pigs fed either a basal diet or GP-supplemented diet. (DOCX)

S3 Fig Alpha diversity rarefaction curves showing (A) trend for increased gut microbial diversity of infected pigs fed the basal diet and (B) no differences in gut microbial diversity of infected pigs fed the GP-supplemented diet.
(DOCX)
