# Supplementary material for: A polyphenol-enriched diet and Ascaris suum infection modulate mucosal immune responses and gut microbiota composition in pigs
Source: PLoS One. 2017 Oct 13;12(10):e0186546. doi: 10.1371/journal.pone.0186546 (PMC5640243; doi:10.1371/journal.pone.0186546)
Supplement: S1 Table — (DOCX) [file pone.0186546.s001.docx]

**Supplementary Table 1.**

Primers sequences and amplicon lengths used in gene expression analysis.

| Gene name | Gene symbol | Sequence (5' to 3') | Amplicon length |
| --- | --- | --- | --- |
| *B2M* | Beta-2-Microglobulin | F: TGAAGCACGTGACTCTCGAT | 70 |
|  |  | R: CTCTGTGATGCCGGTTAGTG |  |
| *CCL2* | Chemokine ligand 2 | F: CTTCTGCACCCAGGTCCTT | 93 |
|  |  | R: CGCTGCATCGAGATCTTCTT |  |
| *CCL17* | Chemokine ligand 17 | F: TCTGTTCTGACCCCAAGGAC | 75 |
|  |  | R: GGTCCTGTGGCTTCATGTTT |  |
| *CCL22* | Chemokine ligand 22 | F: CCCTGCGTGTGGTGAAGTAT | 88 |
|  |  | R: ATCTCTCGGTCCCTCAAGGT |  |
| *CCL26* | Chemokine ligand 26 | F: GCTCCCCAGCTTTTTCTGAT | 100 |
|  |  | R: TGTGGCTGTATTGGAAGCAG |  |
| *IFNG* | Interferon gamma | F:CCATTCAAAGGAGCATGGAT | 76 |
|  |  | R: TTCAGTTTCCCAGAGCTACCA |  |
| *IL8* | Interleukin 8 | F: TTGCCAGAGAAATCACAGGA | 78 |
|  |  | R: TGCATGGGACACTGGAAATA |  |
| *IL12B* | Interleukin 12p40 | F: GACCAGAAAGAGCCCAAAAAC | 70 |
|  |  | R: AGGTGAAACGTCCGGAGTAA |  |
| *IL5* | Interleukin 5 | F: TGCCTACGTTAGTGCCATTG | 82 |
|  |  | R: TCGATGAATGGAGAGCAGTG |  |
| *IL5RA* | Interleukin 5 receptor alpha | F: GGGACTCTGGAGTGAGTGGA | 95 |
|  |  | R: CGGCTGCTGTAAGCACAATA |  |
| *RPL13A* | Ribosomal protein L13a | F: ATTGTGGCCAAGCAGGTACT | 76 |
|  |  | R: AATTGCCAGAAATGTTGATGC |  |
| *TBP* | TATA box binding protein | F: ACGTTCGGTTTAGGTTGCAG | 96 |
|  |  | R: CAGGAACGCTCTGGAGTTCT |  |
