# Supplementary material for: A polyphenol-enriched diet and Ascaris suum infection modulate mucosal immune responses and gut microbiota composition in pigs
Source: PLoS One. 2017 Oct 13;12(10):e0186546. doi: 10.1371/journal.pone.0186546 (PMC5640243; doi:10.1371/journal.pone.0186546)
Supplement: S1 Fig — (DOCX) [file pone.0186546.s002.docx]

**Supplementary Figure 1. Structures of flavan-3-ol monomeric subunits and an example of a tetrameric proanthocyanidin (condensed tannin).**
