# Supplementary material for: A polyphenol-enriched diet and Ascaris suum infection modulate mucosal immune responses and gut microbiota composition in pigs
Source: PLoS One. 2017 Oct 13;12(10):e0186546. doi: 10.1371/journal.pone.0186546 (PMC5640243; doi:10.1371/journal.pone.0186546)
Supplement: S2 Fig — (DOCX) [file pone.0186546.s003.docx]

**Supplementary Figure 2**

Gene expression of *IL5, IL5RA, IL8* and *IFNG* in the jejunal mucosa of *A. suum*-infected pigs fed either a basal diet or GP-supplemented diet.
