# Supplementary material for: A polyphenol-enriched diet and Ascaris suum infection modulate mucosal immune responses and gut microbiota composition in pigs
Source: PLoS One. 2017 Oct 13;12(10):e0186546. doi: 10.1371/journal.pone.0186546 (PMC5640243; doi:10.1371/journal.pone.0186546)
Supplement: S3 Fig — Alpha diversity rarefaction curves showing (A) trend for increased gut microbial diversity of infected pigs fed the basal diet and (B) no differences in gut microbial diversity of infected pigs fed the GP-supplemented diet. (DOCX) [file pone.0186546.s004.docx]

**Supplementary Figure 3**

Alpha diversity rarefaction curves showing (A) trend for increased gut microbial diversity of infected pigs fed the basal diet and (B) no differences in gut microbial diversity of infected pigs fed the GP-supplemented diet.
